# A Treatise on the Medical Properties of Mercury

**Published:** 1782

**Authors:** 


VI. *A Treatise on the Medical Properties of Mercury. By* John Howard, *Surgeon.* 8vo. Longman, *London,* 1782, 120 pages. 3s.

THE author of this essay is a zealous advocate for salivation in the treatment of the *Lues Venerea.* The merits of this method are discussed in the first of the two sections into which which the work is divided. Mr. Howard is of opinion that mercury in order to cure the difeafe in queftion muft neceffarily produce "a melt-"ing down, attenuation, or fingular fpecies of "putrefaction of the animal fluids;" that thefe are to be confidered as its decifive antivenereal effects; that the fingle hinge on which fuccefs turns in every cafe, no matter by what method it be treated, is the change which takes place in the fyftem at the approach of and during fali-vation; that the more violent this affection, *cæteris paribus*, the greater will be the antivenereal power of the remedy; that although this power is certainly in fome inftances to be obtained, even under the clofeft confinement, without a ptyalifm, or at leaft with fo trifling an affection of the mouth as not to deferve the name of fali-vation, yet that this can be no argument againft the great utility of the method under confine-ment, nor againft the propriety of affecting the mouth, as it only fhews that the internal and moft effential effects of the medicine may, and fome-times do follow without any external mark or fymptom, ftrongly denoting the operation of the medicine as a powerful evacuant.—Thefe are principles which we cannot adopt, nor can we be brought to believe, that in order to cure a

 man man of the venereal diſeaſe it is neceſſary to excite a general ſtate of putridity, ſo that the blood ſhall appear broken in its texture, and the urine become of a dark brown colour, depoſiting a ſediment like dirt: and yet theſe are parts of the picture which our author has given us of the effects of mercury adminiſtered under confinement ſo as to ſalivate.

In ſupport of his doctrine on this ſubject he quotes the writings and practice of Sydenham; admirers as we are, however, of that truly great man in many other inſtances, we cannot allow his authority to have any weight in the preſent.

As an argument in favour of the uſe of mercury under confinement, our author obſerves that throughout the whole proceſs of ſalivation, even at the period when the fœtor and proſtration of ſtrength, &c. are at the height, there are latent ſymptoms of ſtrength generated by the ſtimulus of the remedy, combined with but kept under by thoſe of putrefaction; and which do not indeed then appear, but which immediately ſhew themſelves on the ſubſidence of the flux. Hence it is, he thinks, that although a man after ſalivation comes out from his confinement much thinner than he was before, yet he looks well and has an uncommon propenſity to acquire quire ſpeedily his former health and ſpirits; the quick depletion of his veſſels being followed by as ſudden a repletion, ſo that he generally becomes fatter than he was before.—That ſuch an effect as this may and ſometimes does happen after a firſt ſalivation in a young man of ſtrong healthy *ſtamina*, we have no doubt; but we apprehend that the cauſe of it ought to be ſought for in the firmneſs of the patient's conſtitution rather than in any ſtimulating or invigorating property of mercury. If this remedy really poſſeſſed ſuch a power ſucceſſive ſalivations would bring with them freſh acceſſions of ſtrength to the vital powers; but the fact is, at leaſt ſo far as we can collect from our own obſervations on the ſubject, that every repetition of the ſalivating proceſs brings on an increaſed degree of debility.

In a ſucceeding part of the work we find the author very properly cautioning practitioners againſt the ill conſequences which may ariſe from a too liberal uſe of mercury, or from too violent ſalivation. Accidents of this ſort, we are told, are moſt likely to happen in very irritable or inflammable habits.

In the ſecond ſection Mr. Howard treats of the alterative method. The leading principles laid down down by him when ſpeaking of ſalivation he conſiders as applicable to this other mode of treatment. The great diſcriminating circumſtances which he points out are exerciſe and expoſure to the open air; under a regimen ſomewhat leſs ſtimulating and nutritious than in the ordinary habits of living.

The alterative courſe recommended by Mr. Howard differs not materially from that which is in general uſe. If the cure is begun with frictions, he ſeldom uſes a larger quantity for the firſt fortnight or three weeks than ℥ſs. of *Ung. Mercur. fort.* every night. If the patient objects to frictions, he adminiſters ſmall doſes of *Mercur. Crud. cum Balſ. Sulph. ext.* internally. Care is taken to obviate any tendency to a purging by means of opiates, and in the cure by frictions when coſtiveneſs ſupervenes, and there appears to be no danger of a ſore mouth, he recommends an internal mercurial, either *Mercur. Calcin.* or *Calomel*, if they ſit eaſy, if not, the preparation of crude mercury juſt now mentioned.

He obſerves that in the Idioſyncraſy which is with difficulty affected by mercury, if we mean to raiſe what he ſuppoſes to be its proper anti-venereal effect, the doſes muſt be frequently as large as when the medicine is uſed under confinement.

finement. In ſuch habits he ſometimes thinks it neceſſary to apply *Mercur. alcaliz.* by throwing it dry from a paper into the throat, and ſuddenly waſhing it down with water. A cinnabar fumigation to the throat, unleſs there are ſpreading ulcerations on this part, he conſiders as having too powerful an effect on the ſalivary glands to be truſted to.

Practitioners, who think as we do, that a ſoreneſs of the mouth is by no means eſſential to the cure of the lues venerea, will be equally diſpoſed to doubt the utility of a local ſtimulus applied with a view to haſten an affection of the ſalivary glands.

Mr. Howard allows that an alterative courſe, properly conducted, may with great propriety and general ſucceſs, be applied to moſt of the primary, and to many of the ſecondary ſymptoms of the lues venerea; and that in the idioſyncraſy, too prone to ſalivation, it may be adopted perhaps in preference to the method under confinement. On the other hand he contends that there are caſes in which it will prove ineffectual and even injurious. It is injudicious, he obſerves, to truſt to it when a ſymptom is to be treated which is proceeding with great rapidity; and it will be ineffectual, he thinks, when the power power of habit and idiosyncrasy of the patient so far predominate, that no quantity of mercury, however large, can be made to produce what he terms the proper putrefactive consequences. He objects to it likewise in weak habits where there is a constitutional propensity to Phthisis pulmonalis, upon a supposition that in a tedious alterative course a strong action, not of the acute but of the chronic kind, is excited and kept up in the vessels, and of course is more pernicious than salivation.

The work closes with some remarks on the abuse of topical applications or *sedatives* in venereal complaints. Under this head he ranks not only vitriolic and saturnine preparations, but likewise mercurial and caustic medicaments, all of which he observes have a tendency to check and even cure a venereal sore, although the disease of which this sore is a symptom remains unsubdued in the habit. He objects to the cure of a chancre in any case by caustic, and contends that chancre, chancrous excoriation, and venereal bubo, though apparently local affections only, are yet symptoms of a general disease which exists in the habit from the moment of infection.

